# The emergence of *Clostridium thermocellum* as a high utility candidate for consolidated bioprocessing applications

**DOI:** 10.3389/fchem.2014.00066

**Published:** 2014-08-26

**Authors:** Hannah Akinosho, Kelsey Yee, Dan Close, Arthur Ragauskas

**Affiliations:** ^1^School of Chemistry and Biochemistry, Institute of Paper Science and Technology, Georgia Institute of TechnologyAtlanta, GA, USA; ^2^Oak Ridge National Laboratory, BioEnergy Science CenterOak Ridge, TN, USA; ^3^Biosciences Division, Oak Ridge National LaboratoryOak Ridge, TN, USA; ^4^Department of Chemical and Biomolecular Engineering and Department of Forestry, Wildlife, and Fisheries, University of TennesseeKnoxville, TN, USA

**Keywords:** *Clostridium thermocellum*, cellulosic ethanol, consolidated bioprocessing, omics, cellulosome, biomass utilization

## Abstract

First isolated in 1926, *Clostridium thermocellum* has recently received increased attention as a high utility candidate for use in consolidated bioprocessing (CBP) applications. These applications, which seek to process lignocellulosic biomass directly into useful products such as ethanol, are gaining traction as economically feasible routes toward the production of fuel and other high value chemical compounds as the shortcomings of fossil fuels become evident. This review evaluates *C. thermocellum*'s role in this transitory process by highlighting recent discoveries relating to its genomic, transcriptomic, proteomic, and metabolomic responses to varying biomass sources, with a special emphasis placed on providing an overview of its unique, multivariate enzyme cellulosome complex and the role that this structure performs during biomass degradation. Both naturally evolved and genetically engineered strains are examined in light of their unique attributes and responses to various biomass treatment conditions, and the genetic tools that have been employed for their creation are presented. Several future routes for potential industrial usage are presented, and it is concluded that, although there have been many advances to significantly improve *C. thermocellum*'s amenability to industrial use, several hurdles still remain to be overcome as this unique organism enjoys increased attention within the scientific community.

## Introduction

The current, non-renewable fossil fuels that supply the vast majority of energy needed for transportation will inevitably increase in cost as their supplies are depleted, and already present significant concerns relating to their longevity and sustainability, energy security, and environmental impact. For these reasons, renewable energy sources are attracting considerable attention as alternatives to their non-renewable counterparts. However, in the search for an alternative replacement, any new fuel compound must first meet three primary considerations in order to be regarded as a viable candidate: it must have the potential to supply the world's energy demands, it must be able to reduce negative environmental effects relative to current fossil fuels, and it must be cost-competitive. With the current state of the art, ethanol derived from lignocellulosic biomass addresses two of these considerations, however, its production in a cost-effective manner is currently lacking due to the difficulties in breaking down and converting the sugars locked within the lignocellulosic feedstocks.

**Figure 1:**
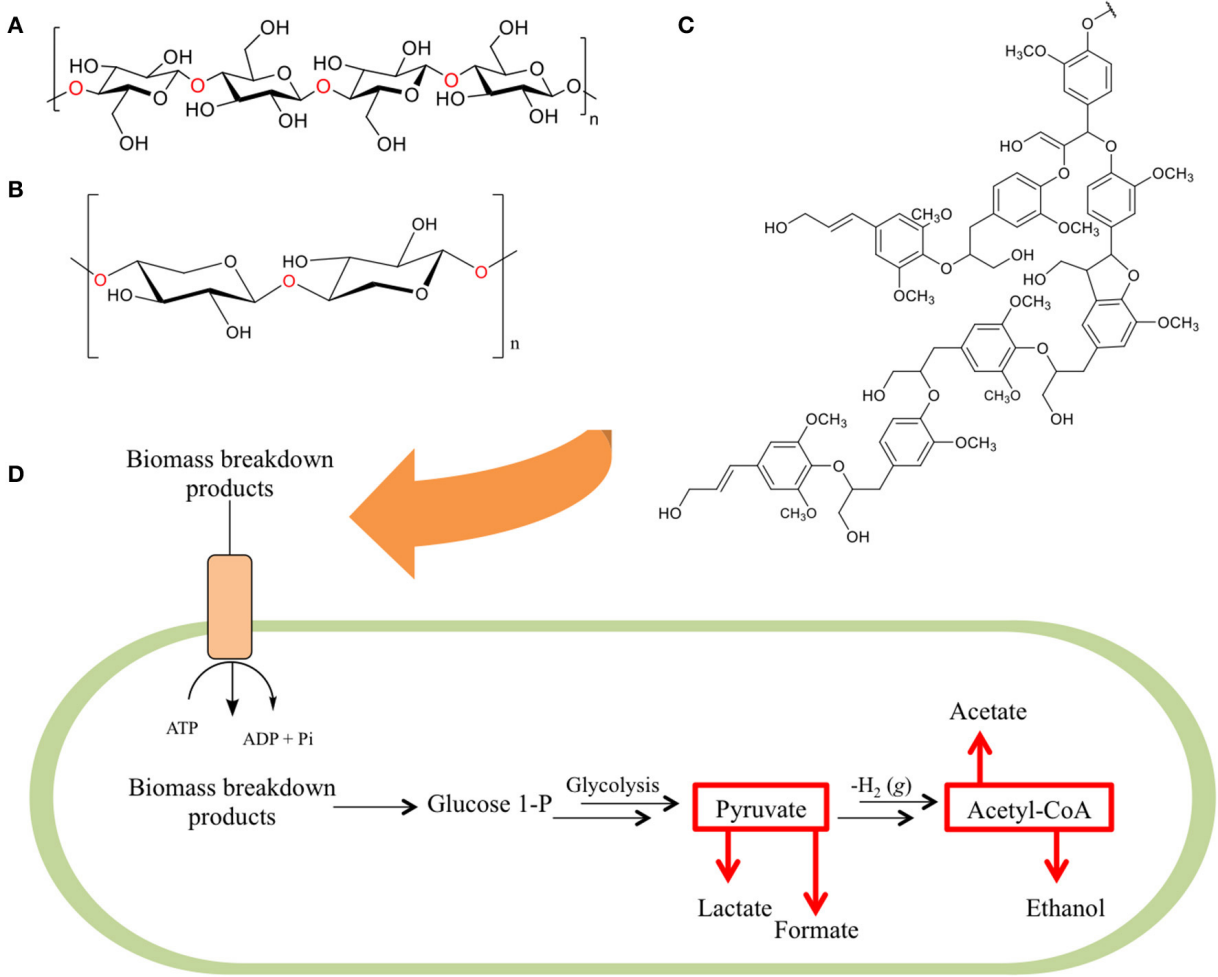
**The three primary constituents of biomass**. Biomass is primarily composed of a combination of **(A)** cellulose—a homopolymer of glucose units, **(B)** hemicellulose (here depicted as xylan—a homopolymer of xylose units), and **(C)** lignin (here depicted as hardwood lignin)—a biopolymer composed of aromatic monomeric units. As these components are degraded **(D)** their fermentable breakdown products are shuttled into bacterial cells via ATP binding cassette transporter proteins and internally converted to glucose-1-phosphate (G1P). G1P is utilized in a modified form of glycolysis that produces pyruvate, which is then broken down into lactate and formate, or converted to acetyl-CoA and further metabolized to acetate and ethanol.

These feedstocks consist primarily of cellulose, hemicellulose, and lignin, collectively referred to as lignocellulose (Figures 1A–C), with smaller contributions consisting of pectin, extractives, and the remaining structural ash. The cellulose component of these mixtures is a linear polymer composed of 7000–15,000 glucose units linked by β-(1-4) glycosidic linkages (Gibson, 2012) arranged into variable repeats of crystalline, paracrystalline, and amorphous regions. The hemicellulose components of lignocellulose, on the other hand, are ~200–400 unit branched or linear polymers comprised of five or six carbon sugars, linked together by glycosidic bonds. The final component, lignin, is a networked polymer composed of phenyl propane units (Zeng, 2013).

One of the major barriers to the microbial production of lignocellulosic ethanol is the conversion of the cellulose and hemicellulose components of biomass to fermentable carbohydrates (Viikari et al., 2012). To overcome this hurdle, several strategies have been proposed including thermal, chemical, biochemical, or microbial approaches, as well as their various combinations, to produce fermentable carbohydrates consisting of either monomeric or polymeric C_6_ and C_5_ sugars. In most process schemes, this conversion of biomass into sugars typically requires an initial pretreatment step to increase plant polysaccharide accessibility, followed by the hydrolytic production of glucose from cellulose, fermentation of the pentose and hexose monomeric sugar streams to ethanol, and distillation of the ethanol from the fermentation mixture (Gupta and Demirbas, 2010). The pretreatment stage of this process is employed to modify the structure of the biomass, increasing accessibility and facilitating improved enzymatic hydrolysis of cellulose. During the hydrolysis stage, acids or hydrolytic enzymes degrade the cellulose into glucose monomers. These acidic treatments, which disrupt the glycosidic linkages in both cellulose (Orozco et al., 2007) and hemicellulose (Lavarack et al., 2002), can further be subdivided into distinct categories depending on the methods employed. In practice, however, most major approaches utilize the application of either concentrated or dilute, ionic-liquid-mediated or solid acids (Amarasekara, 2013).

While this acid-based approach offers lower costs, shorter processing times and greater resistance to product inhibition than hydrolytic enzyme-based approaches, cellulases remain the preferred tools for carrying out hydrolysis. This is because, unlike acid hydrolysis, cellulase-based enzymatic hydrolysis employs milder conditions, reduces capital costs, produces higher yields, and does not generate inhibitory byproducts that can disrupt downstream fermentation by microorganisms (Taherzadeh and Karimi, 2007). In addition, the acid-catalyzed hydrolysis of cellulose generates carbohydrate-derived dehydration products, which are undesirable for the cellulase-based deconstruction of cellulose (Kumar et al., 2013). Recently, studies have been conducted to improve the efficiency and decrease the cost of the enzymatic hydrolysis process using recombinant technologies (Fang and Xia, 2013), ionic liquids (Engel et al., 2012), accessory enzymes (Hu et al., 2011), and alterations of plant cell wall structure focused on modification to their lignin content (Chen and Dixon, 2007; Hisano et al., 2009; Fu et al., 2011; Shen et al., 2013), however, this stage still remains as the main bottleneck preventing cost efficiency. Therefore, as an alternative, the direct saccharification of lignocellulosic biomass has similarly been investigated, but has been shown to negatively impact the efficiency of enzymatic hydrolysis when compared to the saccharification of pretreated substrates in a variety of biomass sources (Intanakul et al., 2003; Zhang et al., 2007a,b).

Currently, most industrial lignocellulosic bioprocessing applications utilize *Escherichia coli*, *Zymomonas mobilis*, *Saccharomyces cerevisiae*, or a handful of other yeast strains in conjunction with exogenous hydrolytic enzymes to release fermentable sugars from the biomass substrate. These organisms, however, are utilized primarily because of their thoroughly developed and studied genetic engineering toolkits, physiology, and metabolic pathways. As a possible exception, *S. cerevisiae* does have several advantageous traits such as its natural ethanol tolerance and ability to grow at acidic pH, however, it remains incapable of surviving at the optimal temperatures of exogenous hydrolytic enzymes and, in its wild type form, is unable to ferment pentose sugars (Vermerris, 2008; Tracy et al., 2012).

One promising approach to circumventing the cost and restriction of this conventional workflow is the use of consolidated bioprocessing (CBP). CBP technologies combine the enzyme production, hydrolysis, and fermentation stages into a single step, improving processing efficiencies, eliminating the need for added exogenous hydrolytic enzymes, and reducing the sugar inhibition of cellulases (Lynd et al., 2005; Xu et al., 2009b; Olson et al., 2012). This approach reduces the number of unit operations, and lowers the overall capital cost of the process (Olson et al., 2010, 2012).

However, for this approach to be economically feasible, an industrially relevant CBP microorganism is required that produces a hydrolytic enzyme system capable of solubilizing a realistic biomass substrate and fermenting both hexose and pentose sugars to ethanol at >90% of its theoretical yield, a titer of at least 40 g/L, and a fermentation rate of >1 g/L/h (Lynd, 1996; Dien et al., 2003). Unfortunately, no microorganisms with these characteristics have yet been discovered, and therefore genetic engineering strategies will be required to develop such a strain. In this regard, two strategies have been developed to engineer an appropriate organism. The first approach seeks to engineer a naturally highly efficient cellulolytic microbe to produce the desired product. The second approach applies a recombinant cellulolytic strategy, and strives to engineer a microbe with naturally high product titer, rate, and yield to express a hydrolytic enzyme system that efficiently solubilizes biomass substrates (Lynd et al., 2005; Alper and Stephanopoulos, 2009; Olson et al., 2012; Blumer-Schuette et al., 2013).

While there are myriad gene sets available that encode enzymes capable of degrading plant biomass, heterologously expressing these suites of enzymes in a non-natively cellulolytic host microorganism requires the transfer, optimization, expression, and coordination of many genes. This potentially represents a more difficult barrier to overcome than engineering a naturally cellulolytic microorganism to produce ethanol. Therefore, thermophilic cellulolytic microorganisms have become attractive targets for this approach, as their growth at high temperatures reduces the risk of contamination, integrates well with existing processing streams, and increases the solubility and digestibility of their required substrates (Demain et al., 2005; Egorova and Antranikian, 2005; Blumer-Schuette et al., 2013). However, regardless of which strategy is realized, each has the potential to unlock an efficient method for the production of ethanol from lignocellulosic biomass (Lynd et al., 2002, 2005; Dien et al., 2003; Zhang, 2011; Olson et al., 2012). To date, a wide variety of microorganisms have been investigated for this process (Taylor et al., 2009; Hasunuma et al., 2013), however, *Clostridium thermocellum* has emerged as a particularly attractive high utility candidate because its use of a cellulosome has demonstrated remarkable enzymatic hydrolysis efficiency compared to free cellulases (Johnson et al., 1982; Lu et al., 2006). This review will therefore focus specifically on *C. thermocellum*'s role as a candidate for CBP and how it can be utilized to improve the suitability of this process toward the production of ethanol as a realistic replacement for existing liquid transportation fuel sources.

## Clostridium thermocellum

### Isolation and initial characterization

*C. thermocellum* is an anaerobic, rod shaped, Gram positive thermophile that is capable of producing ethanol directly from cellulose. Despite its relatively recent rise to popularity in the literature, it was first isolated in 1926 by Viljoen et al. in an attempt to identify novel organisms capable of degrading cellulose. This initial characterization by Viljoen, while basic, provided the framework required for future investigators to work with and develop this unique organism, but proved unreliable due to potential contamination of the culture with additional organisms (Viljoen et al., 1926). The first robust description, therefore, was not available until almost 30 years later. This characterization was the first to report that *C. thermocellum* could grow at temperatures between 50 and 68°C, and demonstrated this growth on cellulose, cellobiose, xylose, and hemicelluloses. It also detailed the major fermentation products, consisting primarily of carbon dioxide and hydrogen gases, formic, acetic, lactic, and succinic acids, and ethanol (McBee, 1954). It is important to note, however, that significant discrepancies in the list of fermentable carbon sources have been shown to exist among alternate characterized *C. thermocellum* strains, so caution must be taken when comparing the growth conditions in the early literature (McBee, 1950).

Following these initial characterizations, there were still many setbacks in the initial attempts at culturing *C. thermocellum* and isolating pure stocks (McBee, 1948). Fortuitously, these have largely been overcome with the development of defined mediums that allow for routine growth and maintenance of *C. thermocellum* cultures (Fleming and Quinn, 1971; Johnson et al., 1981), significantly improving the ease of subculturing and providing an ideal environment for defined selection and genetic modification. As these mediums were developed, they determined a requirement for several essential vitamins, including biotin, pyridoxamine, B_12_, and *p*-aminobenzoic acid (Johnson et al., 1981) and demonstrated a requirement for pH maintenance between 6.2 and 7.7. It is now known, however, that the optimal pH for growth occurs between 6.7 and 7.0 (Freier et al., 1988) and that the optimal growth temperature is 55°C.

Employing these defined growth techniques, *C. thermocellum* can be cultured using either batch or continuous flow approaches, with growth rates of 0.10/h and 0.16/h, respectively (Lynd et al., 1989). However, in the presence of cellulosic material *C. thermocellum* has been observed to form biofilms, which may more closely resemble its growth under environmental conditions. Upon biofilm formation, *C. thermocellum* will orient itself parallel to the carbon fibers of its substrate, forming a single monolayer of cells that will gradually spread outward from the initial site of colonization. These cells will closely mimic the topography of the substrate, with each cell maintaining direct contact if possible (Dumitrache et al., 2013). This orientation may be maintained in order to facilitate the extracellular hydrolysis of the substrate, which is then incorporated into the cell directly as soluble oligosaccharides and used for fermentative catabolism (Zhang and Lynd, 2005). Throughout this process, cells are constantly attaching and detaching from the carbon source, with no apparent correlation to cellular life cycling, and relatively similar percentages of cells involved in division or sporulation in either their attached (11 ± 3%) or detached (5 ± 3%) states (Dumitrache et al., 2013).

One of the main products of this fermentation activity, and indeed the reason that *C. thermocellum* has enjoyed increased attention in the recent past, is ethyl alcohol. However, despite the production of this fermentation end product, wild type *C. thermocellum* can only tolerate ethanol up to 5 g/L before it is significantly inhibited (Herrero and Gomez, 1980). A contributing factor toward this sensitivity has been determined to be the endogenous membrane structure. The predominant lipids that make up *C. thermocellum*'s cell wall are branched and straight chain 16 carbon fatty acids, and 16 carbon plasmalogens that, along with the other components, display a total lipid content of ~82 μ g/mg dry cell weight, with roughly 28% of that weight comprised of plasmogens (Timmons et al., 2009). This membrane orientation leads to a high degree of fluidity that is compounded by the presence of moderate levels of ethanol. As the fluidity increases, the membrane begins to lose its integrity and the health of the cell is negatively impacted. Therefore, in order to tolerate increased levels of ethanol *C. thermocellum* must alter its membrane composition to decrease fluidity and compensate for the artificial fluidity imparted by its own fermentation products.

### Amenability to consolidated bioprocessing

Despite its endogenous disadvantage of ethanol inhibition, *C. thermocellum* retains many qualities that position it well for use as a CBP organism, including its fast rate of digestion of cellulose from plant biomass and its ability to hydrolyze both hemicellulose and cellulose. In addition, it is capable of naturally producing ethanol, albeit at low concentrations (<3 g/L), and one strain, DSM 1313, has both a finished genome sequence and a developed genetic transformation system that allows for the construction of mutant strains (Tyurin et al., 2004; Tripathi et al., 2010; Feinberg et al., 2011; Olson and Lynd, 2012b; Mohr et al., 2013). Although it does suffer from a detriment in that it can only utilize C_6_ sugars, it has been demonstrated to perform efficiently in co-culture with C_6_ and C_5_ utilizing thermophilic anaerobic bacteria, making it an excellent springboard for development into a CBP host.

### Structure, function, and fermentative characteristics of the *C. thermocellum* cellulosome

**Figure 2:**
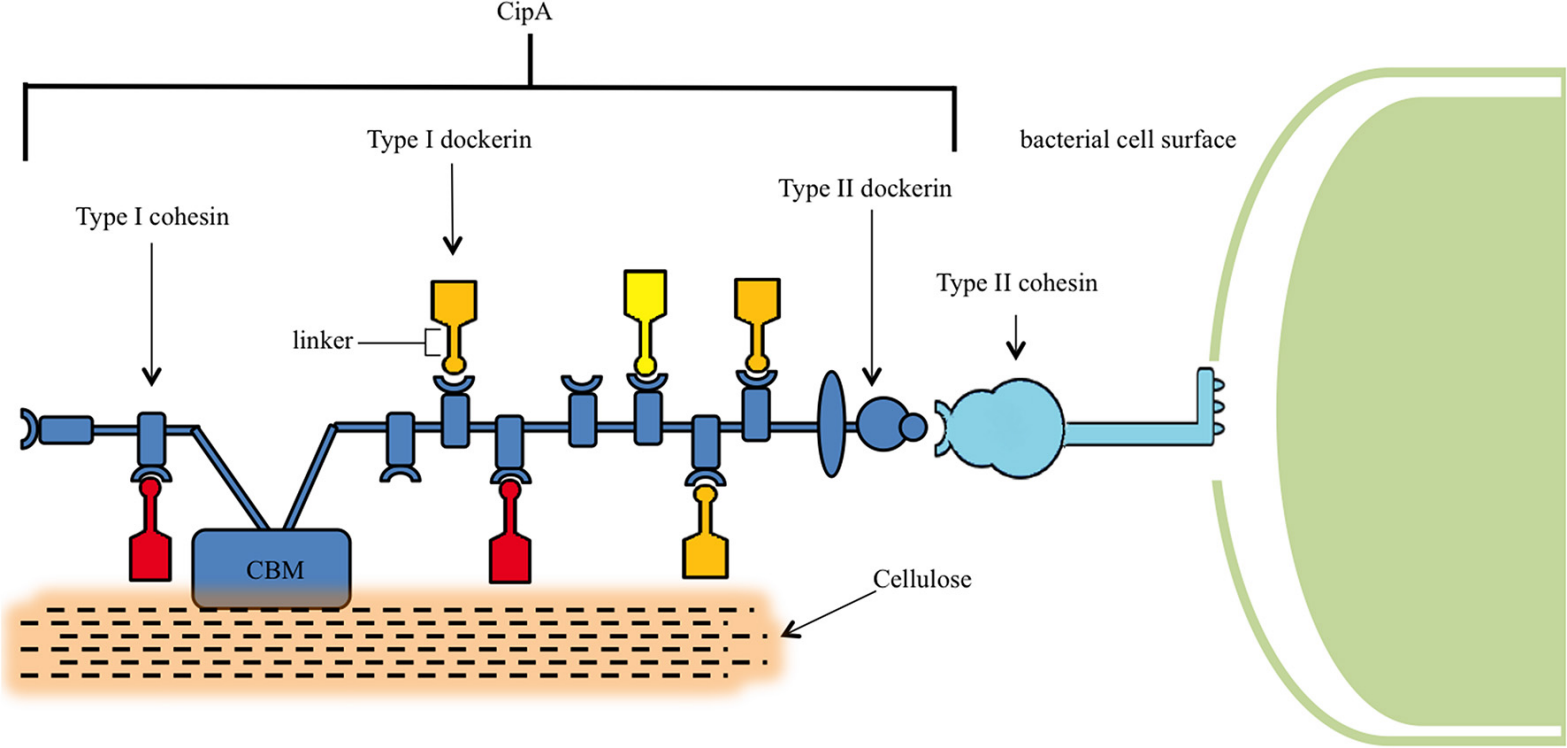
**Structure of the cellulosome**. The central component of the cellulosome, CipA, is bound to the peptidoglycan layer of *C. thermocellum* via binding of the Type II cohesin and Type II dockerin domains. CipA also contains a carbohydrate binding module (CBM), which locates lignocellulose, and Type I cohesins that bind Type I dockerins containing catalytic units for the digestion hemicellulose and cellulose.

The distinguishing feature of *C. thermocellum*, and indeed its most attractive feature as a platform for development into a CBP host, is its cellulosome. The cellulosome is an extracellular multi-enzyme complex 18 nm in diameter with a molecular weight greater than 2 × 10^6^ Da (Uversky and Kataeva, 2006) that is central to *C. thermocellum*'s ability to reduce lignocellulosic biomass recalcitrance (Figure 2) (Bayer et al., 2009). This multi-enzyme complex consists of over 20 distinct enzymes (Wertz and Bédué, 2013), housing cellulases, hemicellulases, pectinases, chitinases, glycosidases, and esterases for the breakdown of lignocellulose (Spinnler et al., 1986; Zverlov et al., 2005a).

Characterization of the cellulosome began in the 1980s, and since that time a stream of discoveries have elucidated its role in cellulose binding (Bayer et al., 1983; Lamed et al., 1983), its position on the bacterial cell wall surface (Bayer et al., 1985), its structure during cellulose degradation (Bayer and Lamed, 1986), and its diversity of associated cellulases (Garcia-Martinez et al., 1980). Central to the assembly of this complex is a macromolecular non-catalytic scaffoldin protein known as CipA. This CipA scaffoldin contains nine type I cohesin domains that bind to type I dockerin domains, which are in turn connected to the catalytic domains of their enzymes through a linker (Dror et al., 2003b) in a calcium-dependent fashion (Shimon et al., 1997). CipA is itself anchored to the bacterial cell surface by way of a type II dockerin and mediated by the LpB, Orf2p, and SdbA anchoring proteins (Dror et al., 2003b) and, in addition, also contains a carbohydrate binding module that attaches the cellulosome to its carbohydrate substrate (Gilbert, 2007).

Crystallographic interrogation has suggested that these integral cohesin-dockerin complexes are primarily mediated by hydrophobic interactions, (Carvalho et al., 2003), and these results have been supported via subsequent molecular dynamics simulations as well (Xu et al., 2009a). As such, it has been presumed that the cellulosome assembles in a non-selective or mildly selective manner due to the inability to assign each dockerin to a single cohesin and the relative similarities in affinity between several dockerins and cohesins (Shimon et al., 1997). However, evidence has recently surfaced that suggests some degree of selectivity. Sakka et al. observed the binding of the CelJ dockerin only to selected cohesin modules, indicating a degree of specificity during cohesin-dockerin recognition that was not previously detected (Sakka et al., 2009). Similarly, Borne et al. have studied the role of randomness during the binding of an alternative *Clostridium cellulolyticum* dockerin to a chimeric scaffoldin containing one *C. cellulolyticum* cohesin and one *C. thermocellum* cohesin. In this case, binding occurred successively in a manner dependent on linker length, reinforcing the notion of order during cellulosomal assembly (Borne et al., 2013).

Findings that support selectivity surrounding enzyme recruitment and/or synergistic effects present during the digestion of biomass point toward major advancements in the production of cellulosic ethanol via the optimization of enzyme combinations. For example, opportunities for synergy between cellulases in *C. thermocellum*'s cellulosome during the degradation of crystalline cellulose increase statistically as the number of cohesins present on the scaffoldin increases. In one study, the inclusion of two cohesins instead of one on the cellulosome increased synergism by a factor of 1.7 (Krauss et al., 2012).

To take advantage of this fact, and leverage the utility of the cellulosome itself, an artificial cellulosome, termed the rosettasome has been genetically engineered to incorporate the dockerin domains of cellulases from *C. thermocellum*. Just as with the native cellulosome, this rosettasome has demonstrated enhanced cellulolytic activities as additional cellulases have been attached (Mitsuzawa et al., 2009). Building upon these efforts, Gefen et al. have developed a chimeric cellulosome, BglA-CohII,that was designed to manage cellobiose inhibition by affixing a β-glucosidase (BGL) to one of the open binding domains. This attachment of BGL lessened cellobiose inhibition in the presence of Avicel and pretreated switchgrass relative to the native cellulosome with or without BGL present (Gefen et al., 2012).

These findings have led to the development of the plasticity theory, which rationalizes this synergistic behavior. This theory contends that the flexibility of a linker within the cellulosome directly leads to its enhanced adaptability toward utilization of different substrates. Coarse-grain models have investigated this theory by monitoring plasticity and uncovering the preferential scaffoldin binding of dockerins from CbhA, a large endoglucanase, over those from the smaller CelS exoglucanase and Cel5B endoglucanase, even though each had the potential to bind to any cohesin. In these models, the large structure of the CelS exoglucanase appeared to influence key parameters such as its extended scaffoldin residence time and its prolonged diffusion rate, both of which improved its likelihood of binding (Bomble et al., 2011).

Regardless of the components employed, the cellulosome breaks down its lignocellulosic substrate into cellodextrins, which are brought into the cell via one of at least five identified ATP binding cassette transporter proteins (Nataf et al., 2009) in order to support a modified form of glycolysis (Gefen et al., 2012). Once within the cell, cellobiose phosphorylase or cellodextrin phosphorylase phosphorylates the cellobiose or cellodextrin, respectively, to yield glucose-1-phosphate and glucose. These compounds are then shunted to the Embden-Meyerhof pathway, and glycolysis takes place to yield pyruvate, GTP, and ATP. Thereafter, a series of phosphorylation reactions follow, although the exact nature and flux of these reactions has not yet been fully elucidated (Zhou et al., 2013). Under our current understanding, both ATP and GTP-linked glucokinases have been identified in *C. thermocellum*, as well as phosphoenolpyruvate carboxykinase, which may be responsible for the conversion of phosphoenolpyruvate to oxaloacetic acid. This has led to the assumption that both of these compounds undergo glycolysis to produce ethanol during fermentation (Zhou et al., 2013). Pyruvate is similarly converted into several fermentation products depending on the enzyme that catalyzes the reaction, with lactate dehydrogenase forming lactate and pyruvate formate-lyase forming formate (Rydzak et al., 2011). These products are then available for use just as with traditional processing strategies, completing the CBP process.

The cellulosome is one of the fastest crystalline cellulose utilizers, however, there are many other hydrolytic enzymes associated with the cellulosome, including pectinases and hemicellulases, which are also essential for digestion of biomass feedstocks. While relatively fewer studies have been undertaken to explore these components, when *C. thermocellum*'s draft genome sequence was screened for open reading frames related to cellulosomal components, it was discovered that only one third of these were related to cellulases and the rest were related to hemicellulases, pectinases, chitinases, glycosidases, and esterases (Zverlov et al., 2005a). Of particular interest from these groups of enzymes are the hemicellulases, which can degrade the hemicellulose matrix through the random cleavage of carbohydrates. Zverlov et al. characterized the structure and activity of two hemicellulytic cellulosome components consisting of xyloglucanase Xgh74A and endoxylanase Xyn10D, demonstrating that when their lysis events occurred in close enough proximity, short oligosaccharides were formed that assisted in exposing the underlying cellulose (Zverlov et al., 2005b). Moreover, it has been demonstrated that *C. thermocellum* JW20 (ATCC 31549) preferentially digests high degree of polymerization xylan, a hemicellulose common to birch wood, with degradation becoming increasingly efficient as the number of monomer units in xylan exceeds six. In contrast, degradation of lower, 2–5 unit, degree of polymerization xylan did not occur until 240 h later and, after 300 h, only xylose remained, as these monomers are not imported by the cell (Wiegel et al., 1985). Taken together, these findings support the hypothesis that *C. thermocellum*'s hemicellulases preferentially degrade high degree of polymerization hemicellulose.

Current studies evaluating the interaction of the cellulosome relative to free cellulases for digestion of either crystalline cellulose or plant biomass have provided additional insights into their mechanisms of hydrolysis, potentially leading to improvements in the deconstruction step through enzyme engineering and optimization of biomass pretreatment conditions. For instance, the cell free cellulosome of *C. thermocellum* can process roughly 40% of high degree of polymerization cellulose (presented as Whatman filter paper) in 120 h, compared with free *Trichoderma reesei* cellulases that can only achieve less than 20% conversion in the same time frame (Resch et al., 2013). However, in contrast, the *T. reesei* free enzyme system was more active on plant biomass than the cell free cellulosome extract. Moreover, post enzymatic hydrolysis images of the crystalline cellulose substrate determined that the mechanisms were vastly different between the free enzyme cocktail, which used a fibril sharpening method, and the cellulosome, which splayed open and separated the individual microfibrils (Resch et al., 2013). Most importantly, however, has been the demonstration of synergistic effects when these two approaches are combined. Ding et al. revealed that this is likely due to a difference in mechanisms between the free enzyme systems and the cellulosome. Using real-time imaging, to show the production of solubilization pits in the surface of the delignified plant biomass treated with free enzyme systems and the splaying of individual microfibrils in cellulosome-treated biomass, they concluded that biomass pretreatments which remove the highest amount of lignin and leave the largest amount of carbohydrates will facilitate improved hydrolysis regardless of whether a free enzyme system or cellulosome is employed (Ding et al., 2012).

### Fermentation of biomass by *C. thermocellum*

The high degree of biomass recalcitrance is one of the major factors limiting the cost-effective production of lignocellulosic ethanol. Therefore, the ability of *C. thermocellum* to efficiently digest a range of biomass structures is an important consideration for its practicality as a CBP host. To investigate its fermentative abilities, Puls et al. compiled one of the earliest characterization studies relating to the solid residuals remaining after cellulosomal processing of steam pretreated, sodium chlorite delignified birchwood by *C. thermocellum*. It was discovered that, following treatment, the solid residuals contained an unchanged crystallinity content (52%) that was attributed to the simultaneous hydrolysis of amorphous and crystalline cellulose. Cellulose experienced an increase in its weight-average degree of polymerization, while the polydispersity remained the same following microbial treatment, indicating the preferential consumption of low degree of polymerization cellulose. These findings ran contrary to those obtained using free cellulases from *Neocallimastix frontalis*, *Trichoderma koningii*, and *Penicillium pinophilum*, providing one of the first indications that the organization and ultrastructure of *C. thermocellum's* cellulosome contained unusual properties (Puls and Wood, 1991).

Since that time, many additional studies have been performed to elucidate the function of *C. thermocellum*'s cellulosome on a variety of substrates. One of the main focal points of these studies has been to determine how *C. thermocellum*'s cellulosome circumvents the inhibition of activity and adsorption that cellulose crystallinity has imparted on many previously characterized fungal cellulases (Hall et al., 2010; Zhao et al., 2012). In this regard, it has been determined that *C. thermocellum* approaches deconstruction atypically, in that it displays a remarkable propensity toward the hydrolysis of crystalline cellulose. For instance, *C. thermocellum* is capable of converting 100% of Avicel, which is 74% crystalline, in 100 h, compared to free cellulases isolated from *T. reesei*, which were only able to consume 50% of Avicel in the same time frame (Resch et al., 2013). While this is encouraging, it should be noted that, in general, Avicel demonstrates excellent conversion properties in comparison to pretreated biomass. Therefore, to expand the scope of this evaluation, Shao et al. further identified differences in *C. thermocellum*'s efficiency during the CBP of Avicel and ammonia fiber expansion pretreated (AFEX) corn stover. While Avicel displayed high conversion rates (>95%) after 24 h when treated with *C. thermocellum*, AFEX pretreated corn stover glucan experienced lower conversion rates (60–70%), even after extended incubation times of 4 days. While the reason for this discrepancy in efficiencies was not elucidated during this study, initial enzyme concentrations and restricted cell growth on AFEX pretreated corn stover were ruled out as possibilities (Shao et al., 2011a).

Along with differences in biomass structure, the employment of differing pretreatment methods have also been shown to influence *C. thermocellum*'s digestion and fermentation efficiency. Hörmeyer et al. investigated the treatment of Avicel, poplar (*Populous tremuloides*), and wheat straw (*Triticum vulgare*) with *C. thermocellum* strain NCIB 10682 using either unpretreated, organosolv (methanol/water), or hydrothermolysis pretreated biomass, and used pH to indicate the extent of cellulose metabolism via acetic acid production. Under this experimental design, hydrothermally-treated poplar produced lower pHs (~6.0–7.0) than unpretreated poplar (~7.4) after 150 min of processing, signifying an increased efficiency in the presence of the hydrothermal substrate (Hörmeyer et al., 1988). Likely, this increase in efficiency can be attributed to the structural changes incurred by the biomass during pretreatment, which led to an increased accessibility of the sugars during digestion while maintaining favorable conditions for growth and enzymatic function (Resch et al., 2013). Alternate strategies for overcoming the recalcitrance barrier, such as altering the plant cell wall structure to be more easily digested by reducing lignin content or altering lignin composition, have also been employed (Chen and Dixon, 2007; Hisano et al., 2009). Fu et al. and Yee et al. demonstrated the feasibility of this approach, showing that a transgenic switchgrass with reduced lignin content and syringyl/guaiacyl (S/G) ratios had improved fermentation yield and required a lower severity pretreatment and less enzyme loading to obtain equivalent yields to their control switchgrass when employing a yeast-based fermentation with exogenous hydrolytic enzymes in a simultaneous saccharification and fermentation (SSF) format. More importantly, they observed that *C. thermocellum* exhibited equivalent or higher fermentation yields than the yeast-based SSF approach, which lead to the hypothesis that the cellulosome is more reactive in a CBP format than a cell-free extract configuration (Fu et al., 2011; Yee et al., 2012). In an alternate approach, Bothun et al. subjected *C. thermocellum* to elevated hydrostatic pressures (7.0 and 17.3 MPa) in a high pressure bioreactor, resulting in a ~100-fold rise in the ethanol:acetate ratio compared to batch cultures at atmospheric pressure. These results were attributed to the enhanced solubility of gaseous fermentation products under their reaction conditions (Bothun et al., 2004), further demonstrating the importance of pretreatment conditions on hydrolysis and fermentation efficiency.

### Genomic, transcriptomic, proteomic, and metabolic responses to ethanol production

Due to its high amenability toward use as a CBP organism, *C. thermocellum* has attracted significant interest in its genomic, transcriptomic, proteomic, and metabolomic profiles and their respective dynamics throughout the CBP process. These evaluations have been performed across a variety of different strains and, taken together, provide crucial insight into how it is able to perform the complex reactions necessary to break down and utilize cellulosic material.

At its most basic level, the genome of the type strain, *C. thermocellum* 27405, consists of 3.8 Mb of DNA arranged as a single chromosome. The average guanine/cytosine (GC) content of the genome is a moderate 38.9%, and 3173 candidate protein encoding genes have been identified via automated analysis (Hauser et al., 2010). In addition to the type strain, sequences for several additional strains have also been elucidated and yielded similar characteristics (Hemme et al., 2010; Feinberg et al., 2011; Brown et al., 2012). Genomic analysis following adaptation to increased ethanol tolerance has indicated several conserved genetic alterations, including changes to glucokinases, aminotransferases, transcriptional regulators, aldehyde/alcohol dehydrogenases, and aspartate carbamoyltransferases. In addition, non-conserved changes have been identified in a variety of membrane proteins as well. Taken together, these genetic changes significantly improved *C. thermocellum*'s ethanol tolerance from ~15 to 50 g/L and improved its utility as a CBP host (Shao et al., 2011b).

While relatively few genetic changes were discovered related to enhanced ethanol tolerance, significantly more transcriptomic alterations have been observed that can provide insight into how *C. thermocellum* responds to changes in substrate availability and ethanol production. Transcriptomic analysis revealed a set of 348 genes that displayed significant variation in their expression levels in response to utilization of either cellulose or cellobiose as a carbon source, or concurrent with changes in growth rate resulting from nutrient availability and population density. Of these 348 genes, 78 demonstrated a significant decrease in expression when cellobiose was provided as a carbon source and 95 were up regulated. Of note is that the majority of these genes contained signal peptides, or were transcriptional regulators, indicating that they are likely involved in the extracellular recruitment and uptake of metabolites, demonstrating *C. thermocellum*'s ability to sense and respond to external cues regarding nutrient availability (Riederer et al., 2011). Similarly, switching from cellobiose to cellulose fermentation elicited changes in the expression of roughly 40% of all genes, with expression profiles generally indicating increased transcription levels for those genes related to energy production, translation, glycolysis, and amino acid, nucleotide, and coenzyme metabolism. Expression of these genes under cellulose utilization was shown to be growth stage dependent, with transcription decreasing as the available cellulose is consumed and transcription of genes encoding for cellular structure and motility, chemotaxis, signal transduction, transcription, and cellulosomal proteins becoming increased, presumably due to an increased necessity to discover alternative carbon sources in accordance with the classic feast-or-famine survival strategy (Raman et al., 2011). When pretreated biomass was supplied in place of cellulose or cellobiose, an even larger number of genes displayed differential regulation. Using pretreated yellow poplar as a model carbon source, 1211 genes were up regulated, and 314 were down regulated compared to growth on cellobiose. Of particular note is that 47 of the 81 recognized cellulosome genes (58%) were up regulated upon yellow poplar-mediated biomass growth, compared with only 4 that showed lower expression levels relative to cellobiose fermentation. In addition to these cellulosome genes, significant up regulation was also observed for genes involved in inorganic ion transport and metabolism, signal transduction, and amino acid transport (Wei et al., 2014). Similar regulation profiles were found, albeit with up regulation of phosphate transport and Resistance-Nodulation-Division (RND) transporters, when pretreated switchgrass was substituted for poplar (Wilson et al., 2013a). Together, these results demonstrate the significant differences that can be imparted when *C. thermocellum* transitions between prepared sugars and raw biomass as carbon sources.

In general, the results obtained from these transcriptomic studies are supported by similar proteomic studies that have directly interrogated protein levels under similar growth conditions. Expression of the core metabolic proteins, as predicted, reveals that they are primarily growth-phase dependent in order to position *C. thermocellum* for the most efficient use of the nutrients on hand under growth and stationary phases, leading to much more consistent expression levels relative to specialized proteins such as those found in the cellulosome. Approximately a quarter of the 144 core metabolic proteins demonstrate only a moderate change in expression as the cells transition from exponential to stationary phase, with several notable exceptions including decreases in the presence of pyruvate synthesis machinery and increases in the prevalence of glycogen metabolism, pyruvate catabolism, and end product synthesis pathway proteins (Rydzak et al., 2012). Much more expression variability has been detected, and indeed much more research has been focused, on the proteins comprising the cellulosome. Unlike the relatively consistent expression of core metabolic proteins, cellulosome proteins demonstrate expression variability in response to changes in carbon source availability. When presented with cellobiose, hemicellulases are the most abundant cellulosome components, with XynA, XynC, XynZ, and XghA up regulated alongside of the endoglucanase CelA and GH5 endoglucanases CelB, CelE, and CelG. Conversely, when presented with cellulose as a carbon source, the GH9 cellulases represented the most abundant group, along with the cell surface anchor protein OlpB and the exoglucanases CelS and CelK (Gold and Martin, 2007). These same trends continue to manifest when pretreated switchgrass is used as a feedstock, with the exoglucanase CelK and the GH9 cellulases further increasing in abundance relative to cellulose fermentation. Notably, under switchgrass utilization the xylanases decrease in prevalence, possibly due to removal of the majority of hemicellulose and reduction of xylan content in the switchgrass following dilute acid pretreatment (Raman et al., 2009). Importantly, it has also been noted that expression of many of the cellulosome proteins is decreased following adaption to increased ethanol tolerance. Indeed, while ethanol tolerant strains can still degrade cellulose, both the rate and extent of this degradation is impaired due to this down regulated expression (Williams et al., 2007).

Compared to the genetic, transcriptomic, and proteomic studies that have been performed, there have been relatively few investigations regarding *C. thermocellum*'s metabolomics under laboratory or natural growth conditions. It is known, however, that, relative to cellobiose, growth on cellulose results in diversion of carbon flow into a transhydrogenase-malate pathway, resulting in increases to available NADPH and GTP supplies. Assimilation of ammonia is also up regulated under these growth conditions, resulting from an increase in the production of glutamate dehydrogenase as *C. thermocellum* repositions itself to produce the biosynthetic intermediates necessary to respond to cellulose utilization (Burton and Martin, 2012). Additional evidence suggests that the end products of this fermentative process can similarly alter metabolic activity as well. As ethanol and lactate collect, H_2_ and acetate yields coordinately increase, while ethanol yields themselves are shown to increase upon accumulation of H_2_, acetate, and lactate (Rydzak et al., 2011). In an effort to improve our knowledge regarding *C. thermocellum* metabolism, and to aid in the development of engineered strains, a flux balance model of *C. thermocellum* metabolism has recently been developed (Roberts et al., 2010) that will hopefully aid in developing this nascent field.

## Efforts to enhance ethanol production from *C. thermocellum*

### Development of engineered strains

In nature, *C. thermocellum's* main ecological function is to degrade cellulose, and in this regard it is one of the fastest crystalline cellulose utilizers. This characteristic has led to a series of studies that have robustly characterized its function in regards to the digestion of plant biomass (Saddler and Chan, 1982; Lynd et al., 1989; Raman et al., 2009; Fu et al., 2011; Shao et al., 2011a; Yee et al., 2012; Wilson et al., 2013a), however, until recently there has been a deficit in our understanding of *C. thermocellum*'s genetic and proteomic functions that have hindered its development as an ideal CBP host. The recent attainment of a finished, annotated genome sequence and an enhanced understanding of its gene and protein expression, in combination with metabolic pathway models, has filled this gap and become essential for the development of targeted genetic engineering strategies and optimization of fermentation conditions that are needed to move forward in strain development. In a wider sense, these aspects have also been crucial for improving the feasibility of CBP as a platform for production of biofuels as well (Stevenson and Weimer, 2005; Lu et al., 2006; Brown et al., 2007; Islam et al., 2009; Raman et al., 2009, 2011; Roberts et al., 2010; Rydzak et al., 2011, 2012; Shao et al., 2011b; Ellis et al., 2012; Li et al., 2012; Wilson et al., 2013a,b).

To this end, several engineered strains have been developed using adapted or directed evolution to improve ethanol or inhibitor tolerance, as these traits have been deemed the most important for industrial applications (Table 1). Linville et al. reported the development of a mutant strain through direct evolution of *C. thermocellum* ATCC 27405 that displayed an enhanced growth rate and tolerance up to 17.5% vol/vol dilute acid pretreated poplar hydrolysate (Linville et al., 2013). Resequencing of the wild type and mutant strains indicated that multiple mutations were responsible for this phenotype, including genes related to cell repair and energy metabolism. Similarly, a wild type *C. thermocellum* culture was adapted through sequential passaging to tolerate 8% wt/vol (80 g/L) ethanol and several analysis were performed to determine the basis of this increased tolerance in the mutant strain, which was designated strain *C. thermocellum* EA. Proteomic analysis of this strain by Williams et al. showed changes in membrane-associated proteins, leading them to hypothesize that the increased tolerance was the result of lower quantities and/or lower incorporation rates of proteins into the membrane, preventing increased fluidity upon ethanol exposure (Williams et al., 2007). Further analysis by Timmons et al. corroborated this hypothesis by observing changes in the fatty acid membrane composition that endowed the mutant strain with increased membrane rigidity, reducing the fluidizing effect of ethanol (Timmons et al., 2009). Recently, Brown et al. resequenced the genome of the mutant strain and, in comparison to the wild type, identified the genetic basis of this tolerance as a mutation in the bifunctional *adhE* gene. This was then confirmed by recreating the mutation in the more genetically tractable DSM 1313 strain (Brown et al., 2011).

**Table 1:** **Natural and engineered *C. thermocellum* stains used for consolidated bioprocessing**.

**Strain**	**Growth conditions**				**Products**						**Efficiency**			**References**
	**Substrate**	**Medium**	**Temp (°C)**	**pH**	**Ethanol (g/L)**	**Acetate (g/L)**	**Lactate (g/L)**	**Propionate (g/L)**	**CO_2_ (g/L)**	**H_2_ (g/L)**	**Carbon Recovery**	**Yield**	**Economic feasibility**	
27405	0.3 % wt/vol Milled Filter Paper	EM	60	7.0	0.80	0.54	ND	ND	ND	ND	ND	ND	Low	Lv and Yu, 2013
	Microcrystalline Cellulose (1% wt/vol)	BM7	60	6.0–7.5	1.09	1.49	2.43	0.25	ND	ND	ND	ND	Low	Tachaapaikoon et al., 2012
	^15^N Cellulose (5 g/L)	MTC	58	6.8	1.34	1.17	ND	ND	ND	ND	ND	0.50 (g/g)	Low	Raman et al., 2009
	^14^N Cellulose (5 g/L)				1.27	1.16						0.49 (g/g)		
	Cellobiose (5 g/L)				1.02	1.43						0.49 (g/g)		
	Z-Trim® (5 g/L)				0.54	0.81						0.45 (g/g)		
	Cellulose-Xylan (5 g/L)				0.62	0.71						0.44 (g/g)		
	Cellulose-Pectin (5 g/L)				0.49	0.69						0.39 (g/g)		
	Celluose-Pectin-Xylan (5 g/L)				0.54	0.60						0.38 (g/g)		
	Pretreated Switchgrass (5 g/L)				0.32	0.60						0.37 (g/g)		
	Pretreated Switchgrass (5 g/L)	MTC	58	6.8	0.20	0.50	ND	ND	ND	ND	ND	ND	Low	Wilson et al., 2013a
	Pretreated Populus(5 g/L)				0.30	0.80							Low	
	Avicel (5 g/L)	MTC	58	7.0	0.83	0.83	ND	ND	ND	ND	ND	ND	Low	Raman et al., 2011
DSM 1313	Cellulose	CM3	60	7.8	0.96	0.75	0.38	ND	1.20	0.04	0.88	ND	Low	Weimer and Zeikus, 1977
	Avicel (19.5 g/L)	MTC	55	7.0	1.32	2.74	2.49	ND	ND	ND	0.72	ND	Low	Argyros et al., 2011
	Cellobiose (5 g/L)	Rich media	55	7.0	0.68	1.10	0.25	ND	ND	ND	ND	ND	Low	Tripathi et al., 2010
	Avicel (5 g/L)				0.70	1.10	0.05							
CS7	0.3 % wt/vol Milled Filter Paper	EM	60	7.0	0.79	0.32	ND	ND	ND	ND	ND	ND	Low	Lv and Yu, 2013
CS8	0.3 % wt/vol Milled Filter Paper				0.83	0.43	ND	ND	ND	ND	ND	ND	Low	Lv and Yu, 2013
S14	Microcrystalline Cellulose (1% wt/vol)	BM7	60	6.5–7.0	1.90	3.72	0.74	1.23	ND	ND	ND	ND	Low	Tachaapaikoon et al., 2012
YS	Cellulose	CM3	60	7.3	1.40	1.90	ND	ND	ND	0.10	ND	ND	Low	Lamed et al., 1988
	Cellobiose				1.20	1.80				0.10				
LQRI	0.4% wt/vol Cellulose	GS	60	7.0	0.71	0.96	0.31	ND	1.33	0.05	≥0.80	ND	Low	Ng et al., 1981
	0.4% wt/vol Glucose				0.75	0.89	0.22		1.21	0.04				
	0.4% wt/vol Cellobiose				0.72	0.74	0.21		1.52	0.06				
	Cellulose	CM3	60	7.3	0.90	2.90	ND	ND	ND	0.20	ND	ND	Low	Lamed et al., 1988
	Cellobiose				0.90	3.00				0.20				
JW20	1% (wt/vol) Cellulose	Minimal Media	58–61	6.1–7.5	0.61	1.21	0.43	ND	2.38	0.14	0.87	ND	Low	Freier et al., 1988
BC1	Cellulose, glucose, sorbitol	ND	67	ND	ND	ND	ND	ND	ND	ND	ND	ND	Low	Koeck et al., 2013
M1570	Avicel (19.5 g/L)	MTC	55	7.0	5.61	0.16	0.11	ND	ND	ND	0.61	ND	Low	Argyros et al., 2011

ND, Not defined in original publication.

Isolation of additional *C. thermocellum* strains is also ongoing, with the novel CS7, CS8, and S14 strains being isolated from compost and bagasse paper sludge, respectively (Tachaapaikoon et al., 2012; Lv and Yu, 2013). Interestingly, when the CS7 and CS8 strains were characterized for growth on crystalline cellulose and cellobiose, in contrast to the majority of *C. thermocellum* strains, neither exhibited any xylanase activity. However, both of these strains demonstrated increased ethanol:acetate ratios and enhanced cellulase activity in comparison to the wild type strain. Strain S14 also proved to be notable, as its cellulosomal glycoside hydrolases provided increased crystalline cellulose degradation rates relative to both the wild type and to strain JW20. In addition, strain S14 was found to tolerate both a higher temperature (70°C) and pH (9.0) than the wild type while consuming a broader range of substrates including sorbitol. However, as of yet, CS7, CS8, and S14 do not have draft genome sequences, which will be crucial for the development of genetic or metabolic engineering approaches in these strains.

Draft or finished genome sequences are, however, currently available for six *C. thermocellum* strains including the wild type (ATCC 27405), YS, LQRI, JW20, BC1, and DSM 1313 (Hemme et al., 2010; Feinberg et al., 2011; Brown et al., 2012; Wilson et al., 2013a). *C. thermocellum* YS was isolated from hot springs at Yellow Stone national park and has been characterized as a highly efficient cellulose utilizer. Notably, it is this strain, in tandem with the adherence-defective mutant *C. thermocellum* AD2 strain, that was used in the studies that reported the initial description of the adherence of *C. thermocellum* to insoluble cellulose substrate and paved the way for the discovery of the cellulosome (Bayer et al., 1983; Lamed et al., 1983). Strain YS has since been leveraged for multiple studies reporting on the digestion of lignocellulosic feedstocks, cell surface interactions, the structure and function of the cellulosome, and transcriptomic evaluations in response to plant biomass hydrolysis (Bayer et al., 1985; Lamed et al., 1988; Poole et al., 1992; Fernandes et al., 1999; Dror et al., 2003a, 2005). *C. thermocellum* JW20 was isolated from a cotton bale in Louisiana and LQRI was isolated from a contaminated culture of strain DSM 1313, which at the time was referred to as LQ8 (Ng and Zeikus, 1981; Ng et al., 1981; Hemme et al., 2010). The growth and physiological properties for each of these strains have since been characterized (Lamed and Zeikus, 1980; Ng et al., 1981), with strain JW20 demonstrating the ability to utilize a spectrum of growth substrates ranging from crystalline cellulose to lignocellulosic feedstocks, including pretreated hardwood, straw, and hay (Freier et al., 1988). Most recently, *C. thermocellum* BC1 was isolated from a compost treatment site in Germany, and a draft genome sequence has been established (Koeck et al., 2013). This strain has exhibited improved cellulose hydrolysis and utilization of a wider range of substrates, including glucose and sorbitol, at a higher temperature (67°C) than the wild type strain. The diversity of unique characteristics demonstrated by these strains, and the important contributions they have made toward improving *C. thermocellum*'s position as a relevant CBP host, highlight the importance of continuing to isolate, characterize, and compare new strains that may have advantageous characteristics for CBP applications.

*C. thermocellum* DSM 1313, previously known as *C. thermocellum* LQ8, represents arguably the most important of the strains discovered to date. First isolated in 1926 by Viljoen et al. from manure or soil (Viljoen et al., 1926), it has been widely studied for its cellulolytic and physiological properties, and has been characterized on cellobiose, crystalline cellulose, and lignocellulosic feedstocks (Weimer and Zeikus, 1977; Wiegel and Dykstra, 1984). However, DSM 1313's high utility comes from the establishment of its draft genome sequence in 2011 and the subsequent development of a genetic system for its transformation that has allowed for the construction of mutant strains (Tyurin et al., 2004; Tripathi et al., 2010; Feinberg et al., 2011; Olson and Lynd, 2012b; Mohr et al., 2013). This ability has allowed investigators to target specific genetic changes within the DSM 1313 background, leading to an unparalleled ability to interrogate the genetic basis for observed phenotypes and to develop strains endowed with specific, engineered functions.

In one such study, comparisons were drawn to previous investigations focusing on the use of proteomic analysis and global gene expression data to enhance understanding of *C. thermocellum's* highly efficient cellulosomal hydrolysis of cellulose and hemicellulose. These initial investigations demonstrated that the catalytic sub-units of the cellulosome were assembled based on their substrate and growth rate (Raman et al., 2009; Wilson et al., 2013a), allowing researchers to create mutant strains of DSM 1313 with knockouts of the *cel48S* gene, that encode an abundant and up regulated cellulase during growth on crystalline cellulose, in order to investigate its role in hydrolysis (Olson et al., 2010). Through the use of this targeted approach, they were able to determine that the deletion of *cel48S* reduced growth rate and specific activity by 2-fold, however, also discovered that it was still able to completely solubilize a 10 g/L loading of Avicel. Without the ability to establish this targeted mutation, it would be difficult, if not impossible, to hypothesize this retention of biomass utilization efficiently in light of such a deleterious mutation. Furthermore, these studies are also important in advancing the creation of designer multi-enzyme complexes for industrial applications (Gold and Martin, 2007; Raman et al., 2009; Fontes and Gilbert, 2010; Olson et al., 2010), as was highlighted by the recent improvement in hydrolysis performance achieved by Gefen et al. through targeted cellulosome engineering, resulting in a three-fold increase in crystalline cellulose hydrolysis and a two-fold improvement for switchgrass hydrolysis (Gefen et al., 2012).

Mutational strain development has also been leveraged to increase ethanol titer and tolerance toward the minimum value of 40 g/L that is required for the economic viability of cellulosic ethanol production (Lynd, 1996; Dien et al., 2003). While wild type *C. thermocellum* strains only produce <3 g/L and are tolerant to <16 g/L of ethanol (Rani et al., 1996; Blumer-Schuette et al., 2013), mutant strains constructed through adapted evolution have shown ethanol tolerance up to 80 g/L, albeit with inconsistent and slow growth, and up to 50 g/L with stable growth (Williams et al., 2007; Brown et al., 2011). To achieve these results, mutant strains of DSM 1313 were constructed with disrupted end product fermentation pathways that altered their natural carbon flow and, conversely, increased their ethanol yield (Argyros et al., 2011; Deng et al., 2013; Mohr et al., 2013; Van Der Veen et al., 2013). These strains were established through mutations in their acetate and lactate pathways (Δ*hpt* Δ*ldh* Δ*pta*), however, once subsequently evolved, they produced contrasting results in their effect on ethanol yield. In one case, no increase in ethanol yield was observed following mutation (Van Der Veen et al., 2013), while in a separate report a 4-fold increase was detected (Argyros et al., 2011). However, in both cases it was hypothesized that the mutations led to a redox imbalance because of the secretion of pyruvate and amino acids into the fermentation broth, low product yields, unsubstantial increases in ethanol, and resulting open carbon balances. In an attempt to reconcile these reports, Mohr et al. used a thermotargetron approach to disrupt the acetate and lactate pathways in place of the homologous recombination approach used by Arygros and van der Veen, resulting in a decrease to lactate and acetate production, a slight increase in ethanol production and a 6-fold increase in pyruvate production (Mohr et al., 2013).

Building upon these studies, Deng et al. noted that pyruvate kinase had not been annotated in the DSM 1313 genome sequence and did not register during enzymatic assays. This led them to the hypothesis that a malate shunt was being used to convert phosphoenol pyruvate to pyruvate (Deng et al., 2013). Leveraging the genetic tractability of DSM 1313, they were able to improve ethanol yield by expressing an exogenous pyruvate kinase and deleting the malic enzyme gene in the lactate and acetate pathway deficient strain. As a result, their novel mutant strain achieved a ~3-fold higher ethanol yield, increased carbon recovery, increased formate production, increased ethanol tolerance, and decreased amino acid secretion relative to the parent strain. The sheer number of mutations and genetic knowledge required to achieve this goal perfectly demonstrates the necessity of obtaining a fundamental understanding of gene expression, regulation, redox state, carbon catabolism, and metabolic modeling, and the prerequisite of establishing a functional genetic manipulation system that must be obtained prior to the development of mutant strains for use in CBP settings (Roberts et al., 2010; Blumer-Schuette et al., 2013).

### Co-culture of *C. thermocellum* with other organisms

In addition to the development and isolation of additional *C. thermocellum* strains, it is worth noting that there are naturally highly efficient cellulolytic consortia and mixed cultures of *C. thermocellum* that can be employed as well. However, significant difficulties exist in engineering these populations toward the production of their desired fermentation products at high yields for industrial applications. Nonetheless, these populations still poses a high value in that they can be mined for novel cellulolytic microorganisms (Haruta et al., 2002; Kato et al., 2004; Izquierdo et al., 2010; Sizova et al., 2011; Li et al., 2012; Zuroff and Curtis, 2012). While consortia and mixed-cultures will not be covered in depth in this review, defined co-cultures containing *C. thermocellum* have previously been studied for the digestion of lignocellulosic biomass (Ng et al., 1981; Le Ruyet et al., 1984; Mori, 1990; Geng et al., 2010; He et al., 2011; Li et al., 2012; Lü et al., 2013) and have recently been reviewed elsewhere (Blumer-Schuette et al., 2013). In general, these co-cultures are utilized due to *C. thermocellum*'s unique ability to hydrolyze hemicellulose and cellulose utilizing only the cellodextrin breakdown products and forgoing the consumption of C_5_ sugars (Zhang and Lynd, 2005; Blumer-Schuette et al., 2008), making it amenable to co-culture with pentose utilizing thermophiles. Notably, the highest ethanol titer yet reported for the fermentation of crystalline cellulose has been obtained under these conditions, with the co-culture of a metabolically engineered *C. thermocellum* and *Thermoanaerobacterium saccarolyticum*. This fermentation achieved ~80% of theoretical ethanol yield at 38 g/L, and was able to keep organic acid concentrations below their detection limits (Argyros et al., 2011), demonstrating the utility of this type of approach.

## Tools for genetic manipulation

The evolution and selection of naturally occurring *C. thermocellum* strains has provided an initial springboard for development of more industrially relevant organisms, however, the full realization of this effort requires targeted development and optimization of specific characteristics that will enable *C. thermocellum* to function synergistically toward the production of fuels and chemicals from cellulosic biomass. While development of the tools required for the genetic manipulation of *C. thermocellum* is still in its infancy, significant strides have already been made to enable the introduction of exogenous DNA and selection of successfully modified strains. The utilization and expansion of these efforts will be key to achieving *C. thermocellum*'s full potential as a CBP host.

### Methods for introducing foreign DNA

The primary method for introducing DNA into *C. thermocellum* has been through electroporation. This method, which transiently applies an electrical field to generate openings on the cell surface for the introduction of DNA, has been successfully demonstrated for several available strains (ATCC 27405, DSM 1313, and DSM 4150) and has been optimized specifically for strain DSM 1313 (Tyurin et al., 2004). Particularly of note for the application of this technique to *C. thermocellum* transformation is the relationship between current oscillation and transfection efficiency. Tyurin et al. have demonstrated a one-to-one correspondence between the presence of 24 MHz oscillations and successful transformations, noting that the proper oscillations can be achieved by using a >12 kV/cm field strength during transformation. This field strength was itself noted to contribute significantly to transformation optimization as well, with increasing field strengths up to 25 kV/cm producing higher efficiencies (Tyurin et al., 2005). Using this technique, it has been possible both to present exogenous genes for expression and to introduce genetic modification systems capable of altering the native *C. thermocellum* genome and knocking out endogenous loci (Olson and Lynd, 2012b).

### Genetic delivery systems

Three basic strategies exist for the genetic modification of *C. thermocellum*. The first of these simply places additional genetic material into the organism for expression, ideally adding functionality or complimenting a deficiency in order to better prepare the organism for its intended task. Under this strategy, plasmid DNA is introduced using the electroporation approach discussed above. Depending upon the design of the introduced vector, the gene of interest is then either expressed directly from the plasmid or incorporated into the host genome and replicated along with the endogenous DNA during routine cell maintenance. For plasmid-based expression, in addition to the gene of interest, the plasmid must also contain an origin of replication and a selectable marker. There are several selection markers available (discussed below) but, in general, the thermophilic RepB origin of replication is the most prevalent for use in *C. thermocellum*. This origin, which works via rolling circle replication, has also been synthetically modified to generate a temperature sensitive variant that cannot function above 55°C. This provides an additional layer of flexibility that can be utilized for controllable expression of the novel DNA sequence being added (Olson and Lynd, 2012a). It is also possible to integrate the target DNA sequence directly into the genome through the incorporation of homologous loci up- and downstream of the gene of interest. Under this design, once the construct is successfully introduced the homologous regions can permit recombination for the gene of interest into the *C. thermocellum* genome. This forgoes the need to maintain an additional plasmid within the host, but requires the remaining plasmid DNA to be cured following genetic introduction. Either of these two approaches is equally acceptable, and their utilization is usually made on a case-by-case basis following careful assessment of the experimental design.

**Figure 3:**
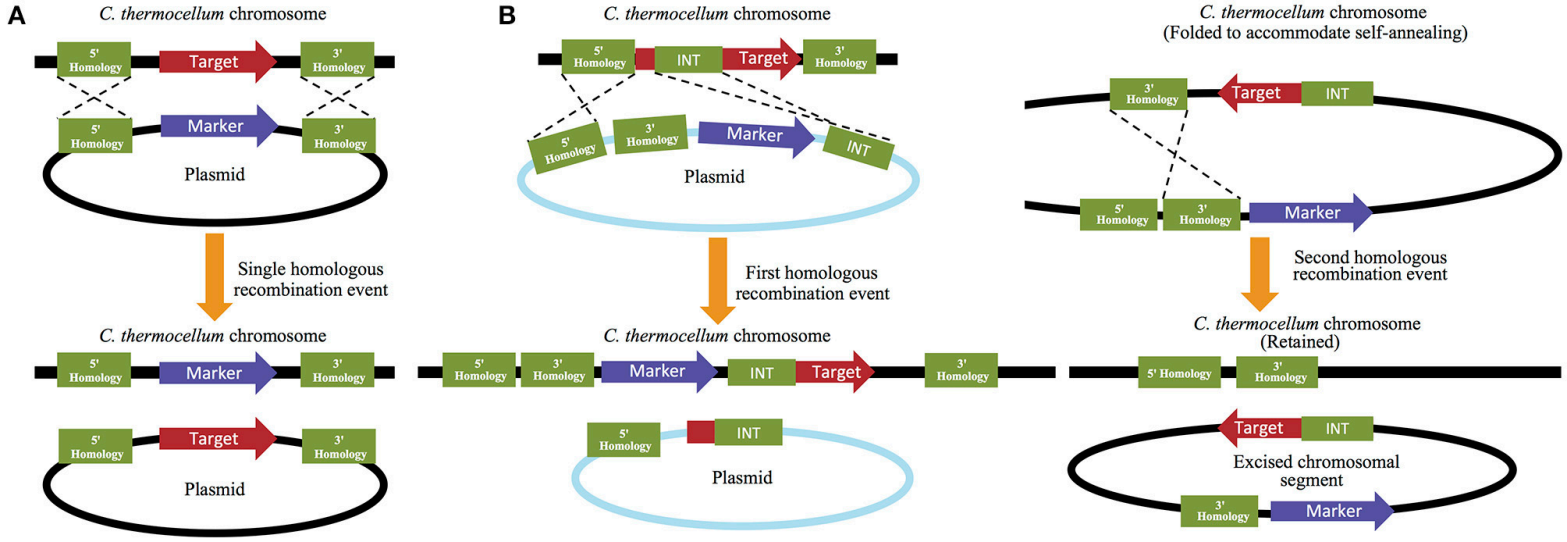
**Targeted gene deletion in *C. thermocellum***. Targeted gene deletions that **(A)** retain their selection markers can be performed quickly using only a single homologous recombination step, while strategies that **(B)** remove the marker and allow it to be reused for subsequent genetic manipulations require multiple rounds of homologous recombination and selection.

The second system performs the opposite function by permitting the removal of endogenous genes from the *C. thermocellum* genome. This plasmid-based strategy can be performed either by replacing the targeted deletion gene with a selectable marker, or by a multi-step process that allows for gene removal followed by marker removal. While the former is a much quicker process, the latter allows for the recycling of selectable markers and therefore permits additional downstream modifications to occur (Figure 3). For retention of the selective maker, 5′ and 3′ flanking regions that match 500–100 bp of the 5′ and 3′ flanking regions of the deletion target are designed and placed up- and downstream of the marker. The 5′ flanking region/selective maker/3′ flanking region cassette is then introduced into the cell where the selective maker is homologously recombined in place of the target gene (Olson and Lynd, 2012b). For marker free gene removal, the 5′ and 3′ flanking regions are both placed upstream of the *cat* and *hpt* selection makers (described in detail below) and a third region, which is referred to as the “int region” and is homologous to a 500–1000 bp region of the gene of interest, is placed downstream of the selection markers. In this multi-step process, an initial selection is performed to isolate strains that have achieved homologous recombination at the 5′ flanking and int regions, which successfully replaces a portion of the gene of interest with the remaining 3′ flanking region and *cat* and *hpt* selection markers. A second selection is then made to remove these markers (and the remaining portion of the gene of interest) and isolate the subset of strains that have performed a second homologous recombination event between the two 3′ flaking regions that are now present on the chromosome. This second recombination will successfully remove all exogenous material, leaving only the 5′ and 3′ flanking regions on the chromosome, with no genetic material between them (Argyros et al., 2011). Because this method allows the selective makers to be reused, it is often utilized over the alternative method, despite its additional investment in time and resources.

The third, and newest of the approaches, leverages the function of a mobile group II intron, often referred to as a “targetron,” from *Thermosynechococcus elongatus* to knock out expression of an endogenous gene via the insertion of a non-coding intron into the native sequence. The advantage of this strategy is that the intron can be engineered by the researcher to insert at any desired location within the genome by including short, homologous sequences flanking the intron that will be used to direct it to its intended location within the genome. In addition to these regions of homology, an intron encoded reverse transciptase protein is required that aids in locally melting the target region of the chromosome and facilitating the insertion of the intron sequence. Fortuitously, because of the thermophilic temperatures present during the culture of *C. thermocellum*, the function of this secondary protein product is minimized and the homology of the targeting regions becomes the most important factor regulating insertion efficiency. The plasmid containing these required sequences is incorporated into the organism using standard electroporation techniques, but then does not require any additional cofactors in order to function. When deployed in *C. thermocellum*, this approach was able to knock out six chromosomal genes with efficiencies ranging from 67 to 100%, resulting in the development of a lactate dehydrogenase deficient strain with increased ethanol production (Mohr et al., 2013). The development of this system to function in thermophilic bacteria, and *C. thermocellum* in particular, is a promising development that will hopefully significantly improve the ease with which mutant strains can be developed.

### Selection of modified strains

A key component of any genetic modification strategy is the ability to select for the resulting altered strain at the conclusion of the procedure. Although not nearly as many makers are available as are for mesophilic bacteria such as *E. coli*, a host of selection markers are available and have been validated in *C. thermocellum*. For negative selection, expression of the Thymidine kinase (*tdk*) or Hygromycin phospotransferase (*hpt*) markers may be used to provide resistance against 5-fluorodeoxyuridine and hygromycin, respectively. Of these, *tkd* is often preferred since *C. thermocellum* has an endogenous *hpt* homolog, and thus requires an *hpt* deficient genetic background for proper function (Olson and Lynd, 2012b). The chloramphenicol acetyltransferase (*cat*) and aminoglycoside phosphotransferase (*neo*) markers can be similarly employed for positive selection, however, the former is preferred as the latter has been demonstrated to inhibit growth at the expression levels required for selection (Olson et al., 2010). An additional maker, orotidine 5-phosphate decarboxylase (*pyrF*) can also act as either a positive or negative selection maker. On the one hand, expressing the *pyrF* gene in a *pyrF* deficient host makes it possible to compliment a uracil auxotroph and select only for strains actively expressing the maker. On the other hand, treatment of *pyrF*-expressing strains with 5-fluoroorotic acid will lead to cellular death as those harboring the gene will incorporate it as a toxic uracil analog (Tripathi et al., 2010). Used together, these markers allow researchers to select and modify strains in efforts to further engineer *C. thermocellum* for the optimized production of high value products.

## Future directions

Although ethanol has been the focus of this review, *C. thermocellum* produces several additional fermentation products that may have value in a variety of industries. The production optimization of these pathways can serve to position *C. thermocellum* as a key industrial organism on par with existing models such as *S. cerevisiae*. One potential route for initial optimization is the production of hydrogen, which can serve as a potential energy source for combustion engines or fuel cells when produced in sufficient quantities. Five strains of *C. thermocellum* (1237, 1313, 2360, 4150, and 7072) have already been evaluated to assess their efficiencies in hydrogen production after using microcrystalline cellulose as a feedstock. Under these conditions, yields ranged between 0.7 and 1.2 mol of hydrogen per mol of glucose (Cheng and Liu, 2011). Acknowledging this potential for hydrogen generation, additional recent studies have investigated the steps involved in *C. thermocellum*'s hydrogen synthesis pathways (Carere et al., 2008) and evaluated inclusion of an electrohydrogenesis stage (Lalaurette et al., 2009) to boost hydrogen production.

In addition to hydrogen, lignocellulosic biomass remains an attractive starting material for the production of lactic acid, formic acid, and acetic acid using *C. thermocellum*'s natural fermentation pathways. By mimicking the action of existing lactic acid bacteria or the fungus *Rhizopus oryzae*, which have previously been demonstrated to produce lactate using corn starch biomass (Hou and Shaw, 2008), it would be possible to assemble the basic units for a variety of high value bio-based polymers. Similarly, while methanol carbonylation is currently used to synthesize the majority of acetic acid (Acton, 2013), this process could also be offloaded to *C. thermocellum* under the appropriate CBP conditions. Regardless, the success of these processes will rely heavily on several factors, such as the existing limitation regarding lactic acid (Cheng and Liu, 2011) and formic acid (Sparling et al., 2006) yields, which are currently inversely related to hydrogen production.

Similarly, the production of butanol from lignocellulosic biomass using a CBP platform is another attractive option because butanol, which is more similar to gasoline than ethanol, has a higher energy density, and can be mixed with gasoline at higher ratios. Unfortunately, all *Clostridium* spp that naturally produce butanol are non-cellulolytic, and only two, *Clostridium acetobutylicum* and *Clostridium beijerinckii*, have been studied in detail (Gheshlaghi et al., 2009). Moving toward this goal of butanol production, there have been a series of studies utilizing co-cultures of *C. thermocellum*, and it has recently been reported that a co-culture of *C. thermocellum* and *Clostridium saccharoperbutylacetonicum* N1-4 can produce up to 7.9 g/L butanol in 9 days using crystalline cellulose as a carbon source (Nakayama et al., 2011). Moreover, the recent development of a transformation system for *C. thermocellum* has led to research efforts aimed at engineering *C. thermocellum* with new pathways to produce butanol as well (Kastelowitz et al., 2014). Through these, and other related pathway studies, it may one day be possible to shift all of *C. thermocellum's* natural array of products toward industrial scale production.

### Conflict of interest statement

The authors declare that the research was conducted in the absence of any commercial or financial relationships that could be construed as a potential conflict of interest.
